# Circulating microRNAs and Molecular Oxidative Stress in Older Adults with Neuroprogression Disorders

**DOI:** 10.1155/2021/4409212

**Published:** 2021-10-22

**Authors:** Hadeel A. Al-Rawaf, Ahmad H. Alghadir, Sami A. Gabr

**Affiliations:** ^1^Department of Clinical Laboratory Sciences, College of Applied Medical Sciences, King Saud University, Riyadh, Saudi Arabia; ^2^Department of Rehabilitation Sciences, College of Applied Medical Sciences, King Saud University, Riyadh, Saudi Arabia

## Abstract

**Background:**

circulating microRNAs are potential blood biomarkers differentially expressed in many diseases including neuro depression disorders. It controls the expression of human genes and associated cellular and physiological processes in normal and diseased cells. We aimed to evaluate the potential role of circulating miRNAs and their association with both stress hormones and cellular oxidative stress in neuro depression disorders occurred among older adults.

**Methods:**

a total of 70 healthy subjects were included in this study. Based upon the profile of mood states (POMS-32 score), the participants classified into two groups; healthy subjects (n =30) and depression (n =40). The expression of microRNAs; miR-124, miR-34a-5p, miR-135, and miR-451-a and their correlation with cellular oxidative stress parameters; cellular NO, genes of SOD2, CAT and iNOS, and hormones; cortisol and serotonin were estimated by a quantitative real-time RT-PCR, high-performance liquid chromatography, and ELISA Immunoassay techniques, respectively.

**Results:**

depression was reported in 57.14% of the participants. The results showed a significant increase (p =0.01) in the total mood scores, and relative depression domains in older adults with depression compared to healthy controls. The relative expression levels of miR-124, miR-34a-5p significantly increased and the expression levels of miR-135, and miR-451-a significantly decreased in older adults with depression compared to healthy controls. In addition, the levels of cortisol significantly increased and serotonin (5HT) significantly reduced in all participants with depression. Cellular oxidative stress analysis for depressed subjects showed that serum NO levels and the expression of iNO gene significantly increased conversely with a decline in the molecular expression antioxidative genes; SOD2, CAT, respectively. The results showed that cellular oxidative stress parameters correlated positively with depression scores, cortisol, and negatively with cellular serotonin levels. In depressed subjects, the relative expression of microRNAs correlated positively with depression score, NO, iNOS, cortisol, and negatively associated with SOD2, CAT, and serotonin.

**Conclusion:**

The combination of cellular oxidative stress and hormonal levels strongly supports a role for circulating miRNAs; miR-124, miR-34a-5p, miR-135, and miR-451-a in the regulation of depression and mood disorders among older adults. The expressed microRNAs with their related association to cellular oxidative stress and adrenal hormones are a step towards understanding the role of these small RNA molecules in the progression of depression among older adults. Thus, cellular miRNAs might have a prognostic role in the diagnosis and as a target for treatment strategies in depressed subjects.

## 1. Introduction

microRNAs (miRNAs) are non-coding RNAs differentially expressed in many diseases including neuro depression disorders [1–8]. It was shown to be associated with most of basic biological processes especially, cell differentiation, proliferation, and cellaur oxidative streaass, and apoptosis. The proposed controling role of miRNAs performed by suppression of gene expression of over 90% of human genes either by degrading mRNA or repressing mRNA translation via deficient complementary binding to their target mRNAs [9, 10].

Higher expression of cellular microRNAs was reported in neurons and proposed to regulate celluar processes of brain development including cellular neurogenesis, apoptosis, proliferation, and cellular metabolism [4, 10]. Thus, abnormal or dysregulated expression of cellular miRNAs was significantly associated with many neurological and psychiatric diseases [5, 6, 11, 12]. Cellular miRNAs are easily transferred to human body fluids, particularly serum, plasma, and cerebrospinal fluid (CSF). Also, the differntial expression of miRNAs is stable and its association with the status of cells could be easily detected [13]. Thus, in many diseases, microRNAs were the potential blood biomarkers displaying efficiently the magnitude of the disease on physiological and cellular bases [14].

A bnormal cellular expression of oxidative stress genes also showed to be associated with the pathogenesis of many psychiatric disorders, including depression and anxiety [15–17]. The potential effective role of oxidative stress might realted to the high oxygen consumption and lipid-rich constitution of the brain along with lower both levels of essential celluar antioxidants and deficinent in the activities of cellular antioxidant enzymes [18–21].

In addition, adrenal hormonal changes and sedentary lifestyle recently associated with depression in older adults [22]. They showed that optimizing the levels of the hormones of the HPA axis by xercise interventions plays a potential physiological role in the performance of a psychological state among older adults with depression [22]. Celluar adrenal hormones like cortisol are controlled by the activity of the HPA and its release in the plasma depend up on the magnitude of stress particularly in older adults [23–25]. Also, lower levels of celluar serotonin deficiency was significantly mentioned as causative parameter of depression [26–28].

The abnormal expression of miRNAs in body fluids and brain tissues showed to be associated with the magnitude of depression in both patients and animal models [29–31]. Although miRNAs have potential as biomarkers [32, 33], their roles in the depression, association with cellular oxidative stress, and stress hormones not fully elucidated. Thus, the current study aimed to evaluate the potential role of circulating miRNAs and their association with both stress hormones and cellular oxidative stress in neuro depression disorders occured among older adults.

## 2. Materials and Methods

### 2.1. Subjects

**Figure 1:**
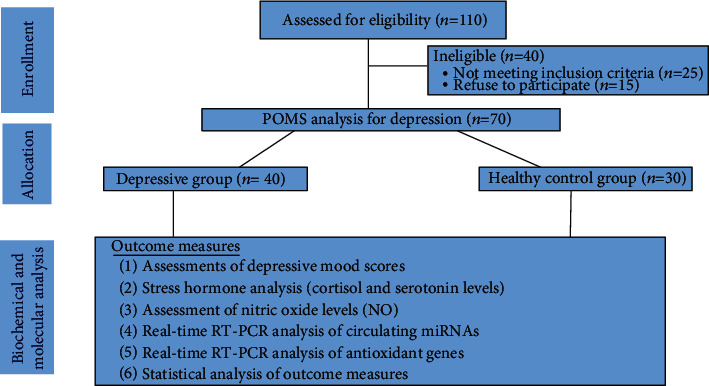
Flow of participants through each stage of the study.

A total of 110 healthy subjects with an age range of 60-85 years old and mean age of (58.3 ± 2.4) were invited via electoral roll randomized selection to participate in this study. Only, 70 subjects who matched the inclusion criteria and agreed to complete the proposed protocol of this study. Subjects with psychiatric illness, chronic infections, severe diseases, abnormalities in endocrine, immune, or even eating habits were excluded from this study. In addition, subjects who taking glucocorticoid medication that affect the measured stress and depressive parameters were excluded from this study. Written informed consent was received from all subjects before participation. Depression scores were evaluated by the profile of mood states (POMS-32) as previously reported [34, 35], and the subjects were classified according to POMS-32 into two groups: the control group (n =30), and the depressive group (n =40) as shown in the flow chart Figure 1. The study protocol was reviewed according to the ethical guidelines of the 1975 Declaration of Helsinki and approved by the ethical committee of RRC, King Saud University, Kingdom of Saudi Arabia, under file number (ID: RRC-2018-018). The demographics and baseline characteristics of participants are shown in Table 1.

### 2.2. Profile of Mood States (POMS)

All participants were subjected to estimate their depressive score by using a pre-validated short version of the POMS as previously reported [34, 35]. In this step mood states of each participant were evaluated by a self-reported questionnaire consists of 32 items (POMS-32 score). For each participant, five different domains of POMS-score; fatigue (6 items), irritation (7 items), vigor (5 items), tension (6 items), and depression (8 items) were used to measure the score depressive mood [34]. In addition, a 5-point Likert scale,^3^ ranging from “not at all” (0) to “extremely” (4) was used to score the POMS-results of each subject. The reliability and validity of this scale are good (*α* = .76–*α* = .95) [35, 36].

In the current study, internal consistency for the different domains ranged from *α* = .86 to *α* = .97, respectively. Based on the POMS analysis, the subjects were classified into two groups control group (n =30), and the depressive group (n =40).

### 2.3. Assessment of Stress Hormones

Saliva and serum samples were collected from each participant according to a proposed time selected for the control of the circadian hormonal range as previously reported [37]. Immunoassay technique was evaluated to estimate cortisol level (pg/mL) of all participants by using the competitive ELISA-kit (Diagnostics Biochem, Ontario, Canada, Inc.) [38]. In addition, serotonin levels (ng/mL) were evaluated in serum samples by using competitive immunoassay ELISA kit (KA1894, Novus Biologicals, Ontario, Canada, Inc.) [38].

### 2.4. Assessment of Nitric Oxide Levels (NO)

Nitric oxide (NO) concentrations serum of all participants. The levels of NO were estimated as nitrate and nitrite at a wavelength of (*λ*; 540 nm) using high-performance liquid chromatography assay as mentioned previously [39] .

### 2.5. Real-Time RT-PCR Analysis of Circulating miRNAs and Antioxidant Genes

#### 2.5.1. Extraction of RNA and Synthesis of *cDNA*

For each participant, the miRNease isolation kit (Qiagen, Hilden, Germany) was used to extract total RNA from serum samples. A reverse-transcription polymerase chain reaction (RTPCR) was applied to analyze total RNA in all serum samples. Then, a complementary DNA (c-DNA) was generated using reverse transcription miScriptII RT kits (Qiagen), and the levels of miRNAs were evaluated by optical density [40].

#### 2.5.2. Real-Time RT-PCR Analysis

Commercial solutions containing the primers and probes for human antioxidant genes; SOD2, CAT, and iNOS (Sigma-Aldrich, Thermo Scientific, Germany) as in Table 1 were used to screen the expression of antioaxidant genes in palsma of all subjects by using a quantitative real-time RT-PCR using an ABI 7300 system (Applied Biosystems) [40]. In addition, the primers of circulating miRNAs; miR-124, miR-34a-5p, miR-135, and miR-451-a (Applied Biosystems, Foster City, CA, U.S.A.) (Table 2), were used to screen the expression of miRNAs in the plasma of all participants by using a quantitative real-time RT-PCR [40]. The average copy number of the resultant PCR components was normalized according to the GAPDH gene which used as an internal housekeeping gene [41]. In PCR process, templets of respective cDNA subjected to four thermal phases; primary denaturation phase (I) (at 94°C for 2 minutes); denaturation phase (II) (at 94°C for 30 seconds); annealing phase (III) (at 59°C for 30 seconds), and amplification phase (IV) (at 72°C for 30 seconds). The PCR phases (II to IV) proceed for 45 cycles and all reactions were measured in triplicated manner [41].

#### 2.5.3. Statistical Analysis

Power calculations of the selected sample size of 70 subjects showed to give an estimated power of 96% and a significance level of 0.05 with an expected frequency of 8.5%.

An SPSS statistical program (SPSS, IBM Statistics V.17) was used to analyze all data produced in this study. The data of continuous variables are expressed as mean ± SD. The frequency differences between the groups was analyzed by using a non-parametric test (Mann–Whitney-Wilcoxon test) and the *χ*2 test, respectively. In all groups, two independent sample t-tests were used for comparison between the studied variables such as depressive mood score (dependent variable), expression levels of miRNAs, antioxidant genes, and stress hormones (independent variables). In addition, multiple stepwise regressions and Pearson's correlations analysis were used to estimate the associations between POMS scores and those of miRNAs levels, stress hormones, antoxidant genes and cellular NO concentrations in older subjects with depression and in controls. All tests were two-tailed; because of multiple assessments, results were only considered statistically significant at a value of p <0.01.

## 3. Results

A total of 70 healthy subjects were recruited to participate in this study (Table 1). They are classified according to depression scores into healthy controls (n =30) and depressive (n =40). Aconsideral increase in BMI (p =0.001), waist (p =0.001), hips (p =0.01), and WHR (p =0.01) as parameters of body composition were considerably increased in adults with depression compared to healthy controls (Table 1). In addition, no influence of lifestyle factors reported among all participants. The results showed that more than 90% of all participants are working with the regular activity of more than 50% (Table 1). Although more than 20% of depressed adults are smokers, the smoking habit has no significant value on depression scores (Table 1).

**Figure 2:**
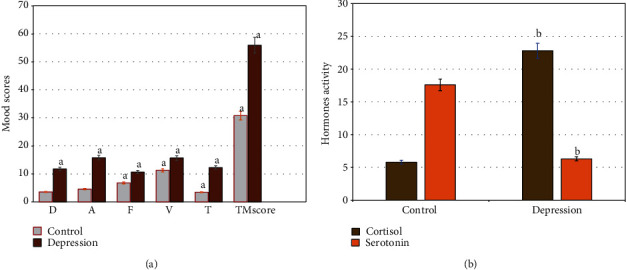
Mood profile scores [A] and the levels of cellular hormones [B] in healthy control and older adults with neurodepression disorders. The results showed significant increase (p =0.01) in the total mood scores, and relative depression domins in older adults with depression compared to healthy controls [A]. In addition, significant increase (p =0.001) in cellular cortisol levels with lower serotonin levels in older adults with depression compared to healthy controls. ^a^ p ≤0.01,^b^ p ≤0.001. D: Depression; A: Anger; F: Fatigue; V: Vigour; T: Tension.

The profile of mood scores among the studied subjects was differentially estimated (Figure 2(a)). All measured mood scores significantly increased in adults with depression (P =0.001) compared to healthy controls. The results showed a significant increase (p =0.01) in the total mood scores, and relative depression domains in older adults with depression compared to healthy controls (Figure 2(a)). In addition, cellular hormonal changes with depression were estimated in all participants (Figure 2(b)). In depressed older adults, a significant increase (p =0.001) in cellular cortisol levels with lower serotonin levels were reported compared to healthy controls (Figure 2(b)). The data support physiological changes in cellular adrenal hormones associated with neuro depression disorders.

**Figure 3:**
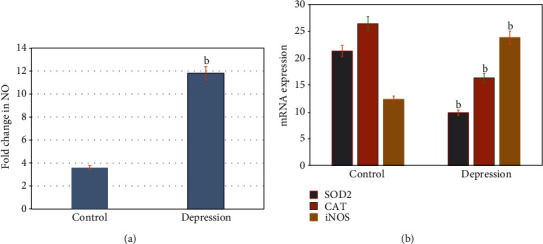
Cellular oxidative profile in healthy control and older adults with neurodepression disorders [A&]. The results showed significant increase (p =0.001) in the levels of celluar nitric oxide (NO) in older adults with depression compared to healthy controls [A]. In addition, older adults with depression showed significant increase (p =0.001) in the expression levels of mRNA of cellular iNOS gene with lower levels of the expressed mRNAs of SOD2 and CAT antioxidant genes compared to that of healthy controls. ^a^ p ≤0.01,^b^ p ≤0.001.

To study the influence of cellular oxidative stress on the status of depression, serum levels of cellular nitric oxide (NO) along with the expression rates of mRNAs of cellular iNOS, SOD2, CAT genes were estimated by high-performance liquid chromatography and real-time PCR analysis (Figure 3). The results showed a significant increase (p =0.001) in the levels of cellular nitric oxide (NO) in older adults with depression compared to healthy controls (Figure 3(a)). In addition, older adults with depression showed a significant increase (p =0.001) in the expression levels of mRNA of cellular iNOS gene with lower levels of the expressed mRNAs of SOD2 and CAT antioxidant genes, respectively, compared to that of healthy controls (Figure 3(b)).

The molecular expression of oxidative stress genes significantly associated with physiological changes ion cellular hormones and mood scores (Table 3). In depressed older adults, cellular expression of oxidative stress genes; iNOS, SOD2, and CAT, and NO correlated positively with the changes in the domains of mood profile scores (depression, anger, fatigue, vigor, tension, TMSore), cortisol, and negatively with cellular serotonin levels as shown in the Table 3.

**Figure 4:**
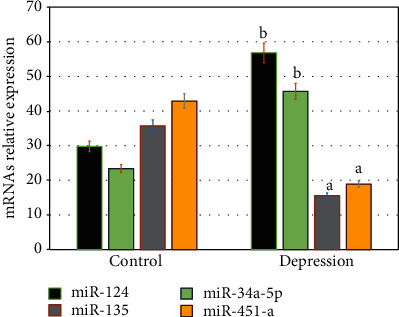
MicroRNAs' differential expression profile in healthy control and older adults with depression.The results showed that the relative expression of miR-124 and miR-34a-5p significantly increased (P =0.001), and miR-135, and miR-451-a significantly reduced (P =0.01) in older adults with depression compared healthy controls. ^a^ p ≤0.01,^b^ p ≤0.001.

In this study, the potential role of cellular micro-RNAs in the pathogenesis and severity of depression was estimated by using and real-time PCR analysis (Figure 4). Differential expression of micro-RNAs; miR-124, miR-34a-5p, miR-135, and miR-451-a was screened in healthy control and older adults with depression (Figure 4). The results showed that the relative expression of miR-124 and miR-34a-5p significantly increased (P =0.001), and miR-135, and miR-451-a significantly reduced (P =0.01) in older adults with depression compared healthy controls (Figure 4). In depressed older adults, miRNAs profiling analysis showed that the relative expression of miR-124, miR-34a-5p, miR-135, and miR-451-a correlated positively with TM-score, NO, iNOS, cortisol, and negatively associated with SOD2, CAT, and serotonin as shown in Table 4. The results showed that cellular miRNAs may have a potential role in the pathogenesis of depression via enhancing molecular expression cellular oxidative stress genes along with an increase of serum nitric oxide. Thus, the estimation of cellular miRNAs might have a prognostic role in the diagnosis and as target for treatment strategies in depressed subjects.

## 4. Discussion

Depression is one of the most mental disorders sowed to be associated with the disease burden worldwide [42]. Recently, symptoms of depression, anxiety, and stress-related disorders have been recognized in more than 32% of patients [43–46].

In this study, depression was reported in 57.14% of the participants. The results showed a significant increase (p =0.01) in the total mood scores, and relative depression domains in older adults with depression compared to healthy controls.

The progression of depression among older adults appear to be influenced with asignificant change or disorders in neurotransmitter systems [22, 25, 26, 28].

The correlation between adrenal cortisol and celluar serotonin was evaluated in this study. A significant increase (p =0.001) in cellular cortisol levels with lower serotonin levels were reported in depressed older adults compared to healthy controls. The data support physiological changes in cellular adrenal hormones in association with neuro depression disordersas previously reported [23–28]. In most studies, the expression and relase of cellular cortisol into blood stream significantly controlled by the magnitude of stress particularly in older adults [23–25]. This stress activates the HPA to further cortisol secrtions into the blood circulation as recently approved [22]. In addition, our results are in line with those who reported significant decline in the levels of blood serotonin in patients with depression [26–28].

In many psychiatric disorders, celluar oxidative stress might be an additional pathogenic factor emrged due to higher oxygen consumption and increased lipid content in the barin of patients [15–20].

Thus, in this study the levels of cellular nitric oxide (NO) and the expression rates of mRNAs of cellular iNOS, SOD2, CAT genes were estimated by using high-performance liquid chromatography and real-time PCR analysis. Cellular nitric oxide (NO), and iNOS gene expression significantly increased and the expression levels of SOD2 and CAT antioxidant genes significantly decreased in older adults with depression. The data showed that oxidative stress pramters; iNOS, SOD2, and CAT, and NO correlated positively with the changes in the domains of mood profile scores (depression, anger, fatigue, vigor, tension, TMSore), cortisol, and negatively with cellular serotonin levels.

In most studies, depression showed to be associated with decline in the plasma concentrations of antioxidants and antioxidative enzyme activities [17, 18, 21]. The deficient in both antioxidant and cellular antioxidant enzymes reduces the protection against the intiated cellular oxidative and nitrogen free radicals which ultimately inducing cellular damage to fatty acids, proteins, DNA, and mitochondria [47–49]. Also, in our recent study, change in the level of adrenal hormones was reported in older adults with higher depressive scores compared to control healthy subjects. In that study, a significant increase in the level cortisol was reported in association with other adrenal hormones such as ACTH and CORT [22].

Previous studies showed that genetic susceptibility may has an additional causatic action in the progression of depression [50, 51]. However, the molecular pathophysiology of depression and its correlation with adrenal hormones and cellular oxidative stress rarly or not fully illustrated. Circulating microRNAs are non coding short strands RNAs showed much intrrerest as gene expression regulators in the pathophysiologies of many diseases [52–56]. The unique non-coding RNA short sequence (23 nucleotides) of microRNAs binds firmly to their targets mRNAs via untranslated region (UTR) producing a suppression or destabilization of genetic code translation which consequently reduce the synthesis or production of associated proteins [57]. microRNAs play a potential role as regulators for cellular physiological activities like cell cycle, apoptosis, neuronal development, and intracellular pathway signaling in normal and devolped diseases, particualry depression [58–61].

In this study, a quantitative real-time PCR (qPCR) was conducted to screen the expression of miRNAs in plasma of all subjects. A relative expression of micro-RNAs; miR-124, miR-34a-5p, miR-135, and miR-451-a was estimated in both healthy subjects and older adults with depression. The results showed that the expression of miR-124 and miR-34a-5p significantly increased (P =0.001), and miR-135, and miR-451-a significantly reduced (P =0.01) in older adults with depression compared to healthy controls.

A quantitative real-time PCR (qPCR) results of previously reported studies confirmed that miR-124 and miRNA-34a-5p expression levels were significantly increased and miR-135 and miRNA-451a expression levels were significantly decreased in patients with depression compared to those expressed in healthy controls [62–64]. The upregulated levels of miR-124 and miRNA-34a-5p and dounregualted levels of miR-135 and miRNA-451a were significantly reported in the plasma of drug-free patients with depression [62–64].

In drug-naive patients, the expression levels of miR-124 and miRNA-34a-5p significantly decreased and those of miR-135 and miRNA-451a significantly increased [65, 66]. This significantly supports the prognostic regulatory role of miRNAs as well as therapeutic targets in treated and non treated patients with depression [66, 67]. In addition, alternation in the expression levels of miRNAs before and after treatment signifies its role in the progression of depression [53, 68].

In this study, the expression of miRNAs was significantly associated with the progression of depression, neurotransmittors, and celluar oxidative stress. In older adults with depression, the expression levels of miR-124, miR-34a-5p, miR-135, and miR-451-a correlated positively with scores of depression, cellular nitric oxide (NO), iNOS, cortisol, and negatively associated with SOD2, CAT, and serotonin.

In normal cells, the presence of miRNAs such as miR-34a-5p significantly regulates cell survival/apoptosis and neuroprotection signaling by controlling a set of cellular genes like as p53, B-cell lymphoma 2 (Bcl-2) and silent information regulator 1 (SIRT1) deacetylase, respectively. miR-34a-5p showed to increase in the expression of p53 via suppression of SIRT1 [69, 70].

In patients with depression, cellular oxidative stress upregulate the expression levels of p53 within cells and consequently increased the expression levels of miR-34a-5p. These results confirmed that oxidative stress increased by depression further enhances the upregulation of miR-34a-5p and miR-124 and downregulation of miR-135, and miR-451-a in the serum and brain tissues of these patients [69, 71]. The overexpressed oxidative free radicals like nitric oxide (NO) significantly contributed to a decrease in the expression levels of cellularSOD2, CAT, and an increase in the expression levels of iNOS [71–73]. Thus, in patients with depression, a correlative physiological relation was reported between higher oxidative stress, lower defensive system, expressed miRNAs, and the severity of the disease.

In patients with depression, the relative expression of cellular miRNAs significantly correlated with excess cortisol activity released from overactived hypothalamic–pituitary–adrenal (HPA) axis and lower levels of serotonin (5HT), respectively. Several astudies reported that both miR-124 and miR-135 significantly regulated by serotonin (5HT) [65, 74–76]. A strong miRNA-target interactions were reported between miR-124 or miR-135 and 5HT transporter and 5HT receptor-1a transcripts. This suggested that higher or lower expression rates of miR-124 or miR-135 significanly associated with serotonin (5HT) levels which consequently colud used as aprognostic factors to measure the response to antidepressant treatment [65, 74–76]. In addition, the expression of miR-34a-5p and miR-451-a significantly correlated with serotonin (5HT) and cortisol levels via affecting of the expression of brain-derived neurotrophic factor (BDNF) expression [67, 77, 78]. In patients with depression, abnormal expression of miRNA-34a and miR-451-a significantly lower the expression of BDNF which affects indirectly on integration of 5HT and COR systems via producing a pro-neuroprotection signaling cascades [79–82].

## 5. Conclusion

The combination of cellular oxidative stress and hormonal levels strongly supports a role for circulating miRNAs; miR-124, miR-34a-5p, miR-135, and miR-451-a in the regulation of depression and mood disorders among older adults. The expressed microRNAs with their related association to cellular oxidative stress and adrenal hormones are a step towards understanding the role of these small RNA molecules in the progression of depression among older adults. Thus, cellular miRNAs might have a prognostic role in the diagnosis and as a target for treatment strategies in depressed subjects.

## Figures and Tables

**Table 1:** The demographics and baseline characteristics of participants.

Parameters	Control group	Depressive group
N	30 (42.86%)	40 (57.14%)
Male/female	23/7	30/10
Age (years)	62.8 ± 3.5	64.7 ± 4.8
BMI (kg/m2)	21.8 ± 3.1	25.1 ± 2.7 ^b^
Waist (cm)	72.6 ± 11.3	96.1 ± 9.6 ^b^
Hips (cm)	85.6 ± 10.2	89.6 ± 21.3 ^a^
WHR	0.85 ± 0.06	1.3 ± 0.12 ^a^
Systolic BP (mmHg)	110.5 ± 6.3	105 ± 11.3
Diastolic BP (mmHg)	73.6 ± 10.4	73.8 ± 10.4
Lifestyle factors, %		
Working	95.5	92.5
Exercising regularly	56.5	52.8
Smoking	12.5	20.9

Values are expressed as mean ± SD; Significance at p <0.05. ^a^ p <0.01, ^b^ p <0.001.

**Table 2:** list of primer sequences used in real-time PCR analysis.

Gene	Primer sequences
*GAPDH*	For 5′-CAA GGT CAT CCA TGA CAA CTT TG-3′ Rev 5′-GTC CAC CAC CCT GTT GCT GTA G-3′
SOD2	For 5′-AGCTGCACCACAGCAAGCAC-3′ Rev 5′-TCCACCACCCTTAGGGCTCA-^3^
CAT	For 5′-TCCGGGATCTTTTTAACGCCATTG-3′ Rev 5′-TCGAGCACGGTAGGGACAGTTCAC-3′
iNOS	For 5′-GTTCCTCAGGCTTGGGTCTT-3′ Rev 5′-TGGGGGAACACAGTAATGGC-3′
MiR-124	For 5′- GTTTCTGTGGTGTAGTCCTCAGGGCTGGGATACTTCTG -3′ Rev 5′- CCAGCCCTGAGGACTACACCACAGAAACAACCTAGCC-3′
miR-34a-5p	For: 5'- GCAGTGGCAGTGTCT TAG-3' Rev: 5'- GGTCCAGTTTTTTTTTTTTTTTACAAC-3'
miR-135	For 5′- ACAUAGGAAUAAAAAGCCAUAtt-3′ Rev 5′- CUAUGGCUUUUUAUUCCUAUGUGA-3′
miR-451a	For 5′- TGGCCGTTACCATTACTGAGTT-3′ Rev 5′- CTCAACTGGTGTCGTGGAGTC-3′

**Table 3:** Correlation between molecular expression of oxidative stress genes with celluar hormones and mood scores in older adults with neurodepression disorders (n =40).

Variables	m-RNA expression of oxidative stress genes in neurodepressed adults (n =40)							
	NO		iNOS		SOD2		CAT	
	Rs	P	Rs	P	Rs	P	Rs	P
Cortisol	0.75	0.01	0.35	0.01	-0.43	0.001	-0.31	0.01
Serotonin	-0.28	0.01	-0.18	0.01	0.15	0.001	0.21	0.01
TMScore	0.56	0.01	0.39	0.01	0.38	0.001	0.46	0.001
Domains of POMScores								
Depression	0.24	0.01	0.14	0.01	0.22	0.01	0.23	0.01
Anger	0.35	0.01	0.23	0.01	0.31	0.01	0.38	0.01
Fatigue	0.13	0.01	0.31	0.01	0.38	0.01	0.34	0.01
Vigour	0.58	0.01	0.14	0.01	0.53	0.01	0.36	0.01
Tension	0.49	0.01	0.18	0.01	0.29	0.01	0.58	0.01

Spearman's rho (rs) of miRNA relative expression levels vs. cellular oxidative stress genes, hormones activity, and mood score, 2-tailed significance (P), and number of subjects are presented. Correlations observed included hormonal markers and mood score frequency based on expression profiles of cellular oxidative stress genes. Nitric oxide (NO); endothelial nitric oxide synthase (iNOS); superoxide dismutase enzyme (SOD2); catalase enzyme (CAT); total mood score (TMscore); Profile of mood states (POMS).

**Table 4:** Correlation studies between microRNA expression, celluar oxidative stress, hormones activity, and mood scores in older adults with neurodepression disorders (n =40).

Variables	MicroRNAs expression in neurodepressed adults (n =40)							
	miR-124		miR-34a-5p		miR-135		miR-451a	
	Rs	P	Rs	P	Rs	P	Rs	P
NO	0.36	0.01	0.42	0.01	0.32	0.001	0.52	0.01
iNOs	0.32	0.001	0.63	0.01	0.39	0.001	0.46	0.01
SOD2	-0.21	0.01	-0.25	0.01	-0.37	0.001	-0.47	0.01
CAT	-0.51	0.01	-0.37	0.01	-0.48	0.001	-0.31	0.01
Cortisol	0.48	0.01	0.28	0.01	0.38	0.001	0.67	0.01
Serotonin	-0.21	0.01	-0.21	0.01	-0.41	0.001	-0.51	0.01
TMscore	0.37	0.004	0.31	0.01	0.75	0.001	0.69	0.01

Spearman's rho (rs) of miRNA relative expression levels vs. cellular oxidative stress, hormones activity, and mood score, 2-tailed significance (P), and number of subjects are presented. Correlations observed included celluar oxidative and hormonal markers, and mood score frequency based on miRNAs profiling. Nitric oxide (NO); endothelial nitric oxide synthase (iNOS); superoxide dismutase enzyme (SOD2); catalase enzyme (CAT); total mood score (TMscore).

## Data Availability

All data generated or analyzed during this study are presented in the manuscript. Please contact the corresponding author for access to data presented in this study.
